# Retrospective Chart Review of Synthetic Cannabinoid Intoxication with Toxicologic Analysis

**DOI:** 10.5811/westjem.2017.12.36968

**Published:** 2018-03-08

**Authors:** Payal Sud, Miles Gordon, Laura Tortora, Matthew Stripp, Damon Borg, Adam Berman

**Affiliations:** * Long Island Jewish Medical Center, Northwell Health, Department of Emergency Medicine, Division of Toxicology, New Hyde Park, New York; † North Shore University Hospital Medical Center, Northwell Health, Department of Emergency Medicine, Division of Toxicology, Manhasset, New York; § Carolinas Medical Center, Department of Emergency Medicine, Charlotte, North Carolina; ¶ Banner-University Medical Center, Department of Emergency Medicine, Department of Toxicology, Phoenix, Arizona; || Glen Cove Hospital, Northwell Health, Department of Emergency Medicine, Glen Cove, New York; ** Cordant Health Solutions, Huntington, New York

## Abstract

**Introduction:**

Use of synthetic cannabinoids (SC) has recently emerged as a new drug epidemic. Our emergency departments (EDs) received a surge of SC users presenting with lethargy and bradycardia, contrasting prior reports of SC-induced tachycardia and agitation. Our goal was to describe these novel presentations and characterize the compounds.

**Methods:**

We present a case series of patients with SC intoxication who presented to our toxicology service covering two tertiary care EDs between 2/11/2015 and 6/23/2015. A retrospective chart review recorded initial vital signs, chief complaint and clinical course. Urine, blood and xenobiotic samples were analyzed using either liquid chromatography/mass spectrometry or gas chromatography/mass spectrometry. We compared resulting spectra against databases containing numerous SCs or metabolites and scored based on a reference comparison.

**Results:**

Between 2/11/2015 and 6/23/2015, we identified 141 visits. Males comprised 139 visits (age range 21–68 years; median 35, interquartile range 20). Sixty-eight percent presented with lethargy or loss of consciousness. Hypotension (SBP <90 mmHg) and bradycardia (HR<60 bpm) were seen in 10% and 24% of visits, respectively. While most patients were discharged after observation, three were admitted to the intensive care unit and seven to telemetry. Admissions were for vital sign instability, bradycardia requiring pacing, prolonged sedation and respiratory failure requiring mechanical ventilation.

Laboratory analysis revealed SC in the XLR-11 family in 18/36 drug, 9/12 blood, and 23/31 urine samples. Carboxamide indazole derivative (CID) family compounds were detected in 13/36 drug samples, 21/31 urine samples, but no blood samples; 11/31 drug samples contained both XLR-11 and CID. Other compounds detected included PB-22 and nicotine. No JWH compounds, opiates, imidazoline receptor agonists, benzodiazepines or other sedative-hypnotics were detected.

**Conclusion:**

Unlike their predecessors, novel SC may be associated with significant central nervous system depression and bradycardia. While prior reports indicated that SC mostly contained JWH compounds, none were detected in these samples. The most commonly identified compounds in this series were CID and alkyl SC derivatives, such as INACA compounds and XLR-11. These tend to be full agonists at the cannabinoid receptor and are presumably more potent. The lack of other depressants suggests that the clinical findings are due to the combination of these compounds and not coingestants or adulterants. SC intoxication should be considered for patients with undifferentiated psychomotor depression and bradycardia.

## INTRODUCTION

Synthetic cannabinoids (SC) are a class of drugs that are becoming increasingly popular throughout the United States and Europe. Also known as “K2,” “spice,” spike,” or “legal marijuana,” SC are causing intoxication requiring emergency department (ED) visits in epidemic and unparalleled numbers.^1^ Patients present with a wide array of symptoms, ranging from nausea and vomiting to confusion, agitation, short-term memory loss, cognitive impairment, psychosis, seizures, arrhythmias, strokes and even death.^2^ SC have often been associated with sympathomimetic effects such as mydriasis, hypertension and tachycardia.^2^ We present a case series of patients with SC intoxication who presented atypically with central nervous system (CNS) and cardiovascular depression over a five-month period; in addition, we present an analysis of blood, urine and SC samples using mass spectrometry. Intoxication with SC products should be considered for patients with undifferentiated psychomotor depression and bradycardia in addition to the excitatory effects previously described.

## BACKGROUND

In early 2015 our suburban, tertiary care EDs experienced a large influx of patients presenting with lethargy and psychomotor depression, often requiring admission to the telemetry or intensive care units and rarely requiring intubation. The patients usually experienced sudden and complete resolution of symptoms after several hours in an obtunded state. Large cohorts of these patients simultaneously presented from a nearby psychiatric center that provided inpatient, outpatient and residential services. The increased volume of intoxications exacerbated ED crowding. Patients later admitted to SC use, and some produced samples of the plant material. Questions arose regarding the potential contamination of these substances with other agents, such as clonidine or digoxin, or whether these presentations were due to newer generation SC.

We selected cases for this series from the toxicology consult service database for patients suspected of SC use. Blood and urine samples were collected from the patients when possible. The unknown drug samples were analyzed and compared to a reference database to identify the compounds present.

## MATERIALS AND METHODS

We included two tertiary care EDs in our case series. In total, 141 ED visits were selected by toxicologists from the consult service database based on abnormal triage vitals, history of SC use or an obtunded mental state upon presentation. Twelve blood and 31 urine samples were collected. The 36 samples of plant material provided by patients were collected and analyzed using liquid chromatography/mass spectrometry and gas chromatography/mass spectrometry (GC/MS). The samples were not correlated with specific patients. This retrospective chart review was approved by an institutional review board.

**Population Health Research Capsule**

What do we already know about this issue?

Synthetic cannabinoid intoxication has emerged as an epidemic, and can present with a wide array of gastrointestinal, neuropsychiatric and cardiovascular symptoms.

What was the research question?

Are bradycardia and central nervous system (CNS) depression associated with novel synthetic cannabinoids, or coingestants or adulterants?

What was the major finding of the study?

Novel synthetic cannabinoids were detected with no coingestants and are associated with CNS depression and bradycardia.

How does this improve population health?

Synthetic cannabinoid intoxication should be considered for patients with undifferentiated CNS depression and bradycardia.

### Standards and Reagents

We purchased chemical reagents, including ethyl acetate, methanol, water, and formic acid from VWR International (Bridgeport, NJ). All solvents were high performance liquid chromatography grade or better.

### Sample Preparation

Samples were extracted with organic solvent and concentrated to isolate any drugs present on the plant material. Briefly, 5 mg aliquots of an unknown plant material, or 100 μL of submitted blood/urine, were transferred to screwtop centrifuge tubes. Two mL of ethyl acetate were added and the samples were thoroughly mixed. Samples were extracted for 10 minutes on a nutating mixer at 24 revolutions per minute. The solvent was transferred to clean test tubes and the extracts were evaporated to dryness under nitrogen at 45°C. Samples were reconstituted in 50 μL methanol and 50 μL 0.1% formic acid in water and transferred to conical autosampler vials for analysis by liquid chromatography time-of-flight (TOF) mass spectrometry. Similarly, samples were reconstituted in 50 μL ethyl acetate for GC/MS confirmation analysis. Biological samples underwent a 20-minute room temperature hydrolysis period prior to liquid-liquid extraction.

### Liquid Chromatography Conditions

We used an Agilent Technologies 1290 liquid chromatograph (LC) equipped with a Zorbax Eclipse Plus C-18 column (2.1mm × 50mm × 1.8μm) for chromatographic separation of the unknown plant material extract. The LC columns were maintained at 50°C in the thermostated column compartment. Mobile phases consisted of 0.1% formic acid in deionized water (A) and 100% methanol (B). The mobile phase flow rate was set at 0.7 mL/min. Initial mobile phase conditions were held at 0%B for 0.5 minutes then increased to 95%B over five minutes. Mobile phase conditions returned to initial starting conditions for a final run time of six minutes.

### Time-of-Flight Mass Spectrometry Conditions

We operated an Agilent Technologies 6230 TOF mass spectrometer with a Jetstream electrospray source in positive ion mode with the following common parameters: nitrogen drying gas temperature 350°C; nitrogen sheath gas temperature 400°C; nitrogen drying gas flow 10 L/min; nitrogen sheath gas flow 11 L/min; nebulizer pressure 45 psi; capillary voltage 4000 V; and nozzle voltage 1000 V. Accurate mass spectra were acquired at a rate of 1 spectra per second over the range of 100 – 1700 m/z.

### TOF Data Analysis

We compared all acquired spectra against the Agilent Technologies Forensic Toxicology PCD Accurate Mass Database of over 7,500 compounds. All spectra were scored based on deviation from expected exact mass assignment (ΔPPM), chromatographic retention time, and peak abundance. Scores greater than 90% match were considered positive. Where available, unknown compounds were confirmed as positive by comparison to a known reference material.

### Gas Chromatography Mass Spectrometry Conditions

We used an Agilent Technologies 7980A series gas chromatograph equipped with an HP-5MS column (30m × 0.25mm × 0.25μm), a 5975C series mass selective detector and a 7693 series autoinjector module for chromatographic separation of the unknown plant material extract. The transfer line temperature was 295°C. The oven program consisted of an equilibration time of 0.5 minutes, initial temperature of 100°C, ramp of 15°C/minute to a final temperature of 325°C. The total run time was 20 minutes. The inlet mode was splitless with a temperature of 265°C and an injection volume of 1μL.

### GC/MS Data Analysis

We compared all acquired spectra against the Scientific Working Group for the Analysis of Seized Drugs (SWGDRUG) database. All spectra were scored based on the search quality of the generated spectrum in comparison to the reference spectrum. We considered search quality scores greater than or equal to 90% positively detected based on chromatographic retention time, and peak abundance. Where available, unknown compounds were confirmed as positive by comparison to a known reference material.

## RESULTS

We identified 141 patient visits from 2/11/2015 to 6/23/2015 (Table 1). Of these patients, 139 (98%) were male with a median age of 35 (range 21–68 years old). Ninety-seven (68%) of the patients presented with lethargy or an altered level of consciousness. A smaller proportion presented with hypotension (systolic blood pressure < 90 mmHg) (10%) or bradycardia (heart rate < 60 bpm) (24%).

We analyzed 36 drug samples (Table 2) and found that the majority of them contained carboxamide indazole derivatives (CID) or XLR-11, an alkyl derivative. Eleven of the samples had both derivative classes detected in the mixture and 14 had no SC identified.

We found that 24 of 31 urine samples tested positive for a SC; 74% of urine samples contained XLR-11, and 35% contained carboxamide indazole derivatives (CID). Nine of the 12 blood samples (75%) contained suspected metabolite of XLR-11. None of the blood samples tested positive for CID. There were no JWH compounds, opioids, imidazoline receptor agonist or sedative-hypnotics detected in any of the material, urine or blood samples.

## DISCUSSION

Hundreds of distinct SC compounds have been identified.^2^ SCs are responsible for a rapidly growing number of presentations to EDs throughout the U.S. in the past several years.^1^ SC use causes intense highs and has become popularized due to accessibility, affordability and limited detectability in common drug screens.^3^ Intoxications often present in clusters due to local distribution of a single product and great variability in the herbal mixtures. One study found a range of 2.3–22.9 mg/g of cannabimimetics in the herbal mixtures.^4^ In addition, SC have been found to be more potent than Δ^9^-THC;^2^ the SC 5F-ADB-PINACA, a CID compound similar to a SC detected in our study, is over 1,000 times more potent than Δ^9^-THC.^5^

In March 2011 the U.S. Department of Justice categorized the five most commonly abused SCs (JWH-018, JWH-073, JWH-200, CP-47,497 and its C8 homolog) as Schedule I drugs under 21 U.S.C.811(h) of the Controlled Substances Act.^6^,^7^,^8^ As local outbreaks continued, the novel compounds (detected in this study) were identified and added to the Controlled Substances Act.

ED visits increased from 11,406 in 2010 to 28,531 in 2011.^9^,^10^ Visits from patients 12–17 years old more than doubled from 3,780 to 7,584, while visits from patients18–20 years old increased from 1,881 to 8,212.^9^,^10^ In 2011, SCs were the second most commonly used drug in the 10^th^ grade and the third most common in eighth grade following marijuana and inhalants.^2^,^11^ Despite the federal ban on SCs that year, there was no decline in frequency of use in high school students the following year. However, use declined in each of the next three years.^11^ Users of SCs vary greatly in both demographics and motivation, but are typically males aged 13–59, most with polydrug use and are found in larger, urban populations.^2^,^12^

SCs are known to interact with the cannabinoid receptors, CB_1_ and CB_2_, leading to changes in levels of multiple neurotransmitters including acetylcholine, dopamine, noradrenaline, glutamine and GABA.^2^ Genetic polymorphisms in enzymes responsible for metabolism of SCs can lead to increased blood levels of the parent compound and prolonged duration of action, and therefore a potential increased risk of adverse events.^10^,^13^ In addition, many SC metabolites retain biological activity.^10^,^13^ Combination of these metabolites with accumulation of the parent drug creates complex pharmacodynamics, especially when the multitude of other compounds typically found within herbal mixtures is considered.

SCs have been reported to exhibit a wide array of effects. CNS effects include psychosis, anxiety, agitation, irritability, memory changes, sedation, confusion and hallucinations,^14^ in addition to lowering the seizure threshold in susceptible individuals.^15^ Reported cardiovascular effects include tachycardia, chest pain, dysrhythmias, myocardial ischemia^13^ and cerebrovascular accident caused by embolisms due to cardiac arrhythmias or reversible cerebral vasoconstriction syndrome.^16^,^17^ In an analysis of a Centers for Disease Control and Prevention report of 3,573 calls to poison control for SC-related adverse events, the most common effects were agitation (35%), tachycardia (29%), drowsiness or lethargy (26%), vomiting (16%), and confusion (4%).^1^

In early 2010, JWH-018 was detected in 100% of SC products. However, as legislation regarding SCs changed in 2010 and 2011, the incidence of JWH-018 decreased, while similar yet compositionally distinct compounds appeared. By the end of 2012, JWH-018 was not detected in samples, and XLR-11 became the most common SC detected,^18^ as exhibited in our sample analysis.

In our case series, CID and alkyl SC derivatives, such as INACA compounds and XLR-11,^19^ were the most commonly detected with no opiates, imidazoline receptor agonists, benzodiazepines or other sedative-hypnotics detected that might explain the atypical presentations. Sixty-one percent of the confiscated products contained a SC and 31% contained both XLR-11 and CID. Seventy-five percent of blood samples and 77% of urine samples tested positive for SC. Unlike their predecessors, novel SC appear to be associated with significant CNS depression and bradycardia. The compounds detected in our case series tended to be full agonists at the cannabinoid receptor and are more potent than Δ9-THC.^20^ The lack of other CNS and cardiovascular depressants suggests that the clinical findings are due to the combination of these compounds and not coingestants or adulterants.

It is important to note that many substances detected in the plant samples were not detected in the blood or urine samples. Some examples include 5-Fluoro-NNEI 2′-naphthyl isomer, 5-fluoropentylindole, NM-2201 and NPB-22. There are multiple explanations for these findings. The patient may have used SC products that were not included in our plant samples and therefore would not be associated with the urine and blood samples. It is also possible that the metabolites of the compound were not in the database or that the level was below the LC TOF detection limits. Furthermore, the metabolite may have been metabolized to a common XLR metabolite that was detected, or the drug had already been eliminated from the body.

## LIMITATIONS

Our case series demonstrates some of the severe effects these novel compounds can cause. However, the study has a number of important limitations. First, the selection of patients was based on the judgment of our ED team and toxicologists based on abnormal vital signs, subjective history from the patient, presentation of decreased mental status and clinical judgment. Many intoxicated patients may have been evaluated and treated without being included in the study. In addition, patients may have had altered mental status for reasons other than SC intoxication and may have been erroneously included in the study because their ED arrival was associated with other patients with SC intoxication. Although there were 141 visits, several patients with recurrent intoxications were included as multiple visits in the study.

The SC samples were provided by patients, but it should not be assumed that the specific sample was necessarily the cause of their intoxication. Furthermore, the samples were collected anonymously, without designation to a specific patient, and therefore we were unable to identify which of the patients presenting with bradycardia tested positive for certain compounds. This significantly diminished our ability to conclude that certain types of SC are associated with more profound presentations of bradycardia and psychomotor depression. Lastly, the majority of the patients presented from a large, nearby psychiatric center. The patients often presented as groups, possibly due to simultaneous drug use with the same sample. This patient population tends to have multiple comorbidities, and members may be taking neuroleptic medications that may increase the opportunity for interactions with the cannabinoids. This is a population with an increased risk of substance use, and therefore the results of our case series cannot necessarily be extrapolated to other populations.

## CONCLUSION

SC products are inexpensive, easily obtained, avoid common drug detection screens and cause a wide array of signs and symptoms. The changing composition of available SCs corresponds to the variability exhibited in patient presentations. SC intoxication should be considered for patients with varied clinical effects, including undifferentiated psychomotor depression, loss of consciousness, hypotension and bradycardia.

## Figures and Tables

**Table 1:** Patients presenting with symptoms of synthetic cannabinoid intoxication.

	Total number	%
Total visits	141	100
Male visits	139	98
Lethargy/LOC	97	68
Hypotension (<90 SBP)	14	10
Bradycardia (<60 HR)	34	24
ICU admissions	4	3
Telemetry admissions	10	7

*LOC,* loss of consciousness; *ICU*, intensive care unit.

**Table 2:** Analyses of samples for presence of synthetic cannabinoids.

Sample (total)	Any SC (%)	XLR-11 (%)	CID (%)	XLR-11 and CID (%)	Nicotine (%)	No SC definitively identified (%)
Drug (36)	22 (61)	18 (50)	13 (36)	11 (31)	5 (14)	14 (39)
Blood (12)	9 (75)	9* (75)	Not detected	Not detected	Not detected	3 (25)
Urine (31)	24 (77)	23* (74)	21 (68)	20 (65)	Not detected	7 (23)

*CID*, carboxamide indazole derivatives, *SC*, synthetic cannabinoids.

* Suspected metabolite of XLR-11 (UR-144 compounds).
